# Ethanolic Extract of Propolis (EEP) Enhances the Apoptosis-Inducing Potential of TRAIL in Cancer Cells

**DOI:** 10.3390/molecules14020738

**Published:** 2009-02-13

**Authors:** Ewelina Szliszka, Zenon P. Czuba, Maciej Domino, Bogdan Mazur, Grzegorz Zydowicz, Wojciech Krol

**Affiliations:** Chair and Department of Microbiology and Immunology, Jordana 19, 41 808 ZabrzeMedical University of Silesia in Katowice, Poland

**Keywords:** Propolis and its phenolic compounds, TRAIL, Cancer cells, Apoptosis, Chemopreventive effect, Antitumor activity.

## Abstract

Ethanolic extract of propolis (EEP) is one of the richest sources of phenolic acids and flavonoids. EEP and its phenolic compounds have been known for various biological activities including immunopotentiation, chemopreventive and antitumor effects. Tumor necrosis factor related apoptosis inducing ligand (TRAIL) is a naturally occurring anticancer agent that preferentially induces apoptosis in cancer cells and is not toxic toward normal cells. We examined the cytotoxic and apoptotic effect of EEP and phenolic compounds identified in propolis in combination with TRAIL on HeLa cancer cells. HeLa cells were resistant to TRAIL-induced apoptosis. Our study demonstrated that EEP and its components significantly sensitize to TRAIL induced death in cancer cells. The percentage of the apoptotic cell after exposure to 50 μg/mL EEP and 100 ng/mL TRAIL increased to 71.10±1.16%. The strongest cytotoxic effect in combination with TRAIL on HeLa cells exhibited apigenin and CAPE at the concentration of 50 μM (58.87±0.75% and 49.59±0.39%, respectively). In this report, we show for the first time that EEP markedly augmented TRAIL mediated apoptosis in cancer cells and confirmed the importance of propolis in chemoprevention of malignant tumors.

## 1. Introduction

Cancer is one of the major public health burdens in Europe and United States of America causing approximately 7 million deaths every year worldwide. More than 11 million people are diagnosed with cancer every year and it is estimated that by 2020 there will be 16 million new cases per year [1]. The epidemiologic findings strongly suggest that cancer rates are influenced by environmental factors which are largely preventable, including diet [2]. Therefore, there is need to develop mechanism-based approaches for the management of cancer. Beside the present major treatment modalities for cancer include surgery, chemotherapy, immunotherapy and radiotherapy for the reduce cancer incidence and mortality rates the great significance have cancer prevention.

Chemoprevention is a rapidly growing area of oncology which focuses on prevention of cancer using naturally occurring or synthetic agents. In addition to inhibiting or delaying the onset of neoplasia by blocking neoplastic inception, chemoprevention plays a role in preventing the development of invasive and metastatic properties in established neoplasm. The term “chemoprevention” was first introduced by Dr Michael Sporn [3,4,5,6]. Chemoprevention of cancer thus differs from cancer treatment in that the goal of this approach is to decrease the rate of cancer incidence and death from cancer through pharmacological interventions relying on prevention rather than cure.

Propolis (bee glue) is a natural resinous product of honey bees. The cancer inhibitory effects of phenolic compounds in propolis have been confirmed on a variety of culture cell lines and animal tumor models [7,8]. Epidemiological and preclinical evidence suggest that phenolic and polyphenolic phytochemicals in propolis possess cancer chemopreventive properties [3,4,9]. This has led to an increased emphasis on cancer prevention strategies in which propolis is used as dietary supplement as the richest source of plant phenolics and polyphenolics.

Several mechanisms contribute to the overall cancer preventive and antitumor effects of propolis and its phenolic components. Further study demonstrated that flavonoids, phenolic acids, as well as EEP inhibit the cancer cell cycle progression, cell proliferation and tumor growth, prevent tumor metastasis, induce cell-cycle arrest and apoptosis [7,8,9,10,11]. 

The role of host immune functions has become increasingly important in our understanding of the mechanisms involved in prevention of malignant diseases. EEP stimulated nonspecific immunity, activated humoral immunity and enhanced cell-mediated immunity [9,12,13,14,15]. The enhancement of host immune function by propolis may be beneficial to cancer chemoprevention. 

Tumor necrosis factor related apoptosis inducing ligand (TRAIL) is a member of TNF superfamily capable of selectively inducing apoptosis in cancer cells with no toxicity against normal tissues. Soluble or expressed on lymphocytes T, macrophages and NK cells molecules TRAIL play an important role in immune surveillance and defense mechanism against tumor cells. The cytotoxic effector functions of those immune cells are important for enabling the immune system to cope efficiently with malignancy. TRAIL induces programmed death in various cancer cells through its interaction with the death-domain containing receptor TRAIL-R1 (death receptor 4 – DR4) and/or TRAIL-R2 (death receptor 5 – DR5) [16].

However, some tumor cells are resistant to TRAIL-mediated cytotoxicity. The decreased expression of death receptors TRAIL-R1 and TRAIL-R2 or increased expression of antiapoptotic protein in cancer cells were involved in TRAIL-resistance [17]. We and others have shown that TRAIL-resistant cancer cells can be sensitized by chemotherapeutic agents, ionizing radiation or dietary polyphenols [18,19,20]. 

**Figure 1:**
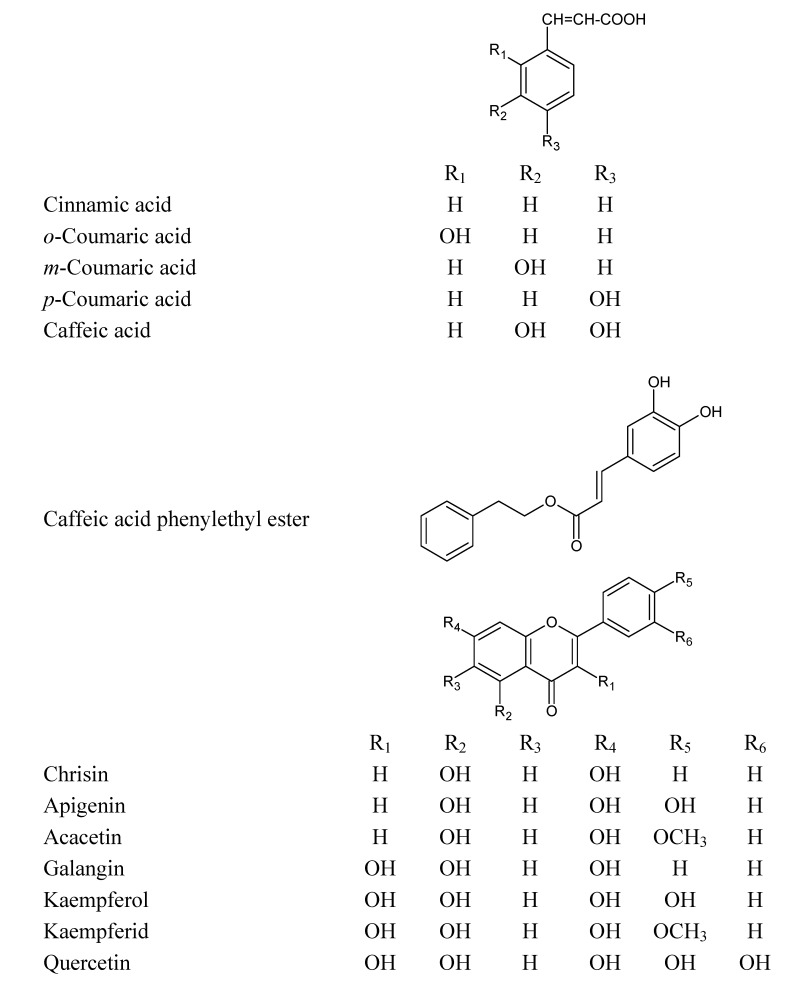
Chemical structures of the phenolic compounds used in this study.

This study was designed to investigate the apoptotic and/or cytotoxic effect of EEP and some of the phenolic compounds found in our sample of propolis [13] (Figure 1) with or without TRAIL on HeLa cells. We showed for the first time that EEP sensitizes HeLa cell to TRAIL induced apoptosis. Our results indicated that EEP markedly augmented TRAIL mediated apoptosis in cancer cells. The overcome of TRAIL-resistance in tumor cells by propolis and its phenolic components may be one of the mechanisms responsible for their cancer chemopreventive effects.

## 2. Results and Discussion

**Figure 2:**
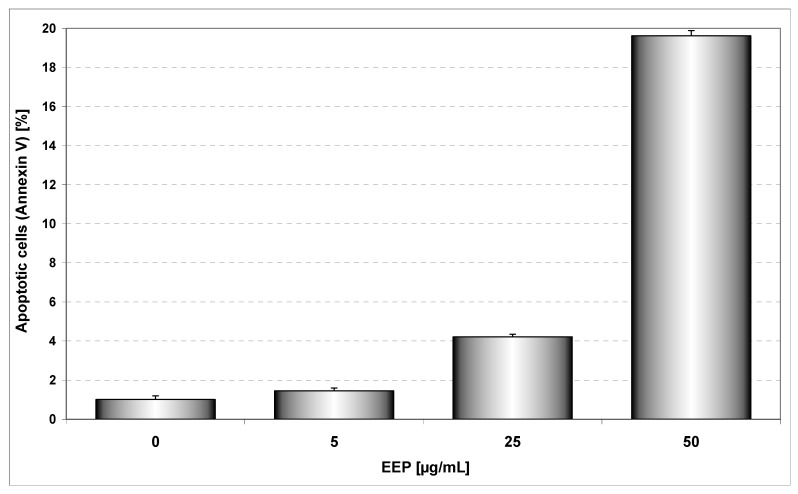
EEP induced apoptosis in HeLa cells. The cancer cells were incubated for 48 hours with EEP at the concentrations of 5 – 50 μg/mL. Detection of apoptotic cell death by annexin V-FITC staining using flow cytometry.

In our search for novel strategies to target tumor cell resistance, we investigated the antitumor effect of the chemopreventive agents, propolis and its phenolic components on the induction of cell death in HeLa cancer cells. EEP has been shown to induce apoptosis in cancer cells *in vitro* and *in vivo* [7,8]. Apoptosis plays a critical role in the pathogenesis of tumor disease. We and others have demonstrated that treatment of cancer cells with propolis inhibited cell proliferation by induced cytotoxicity and apoptosis [7,8,9,11]. EEP inhibited growth and induced apoptosis in HeLa cancer cells in a dose dependent manner. We showed that after incubation for 48 hours, EEP at the concentrations of 5, 25 and 50 μg/mL induced 1.45±0.54%, 4.21±0.5%, 19.62±0.96% cell death, respectively. The annexin V assay revealed apoptotic cells exposed to EEP (Figure 2).

The major active components of propolis are flavonoids and phenolic acids present in EEP at the levels of 25–30%; they have many biological and pharmacological activities including immunopotentiation and antitumor effects [8,9,10,13,21].

**Figure 3:**
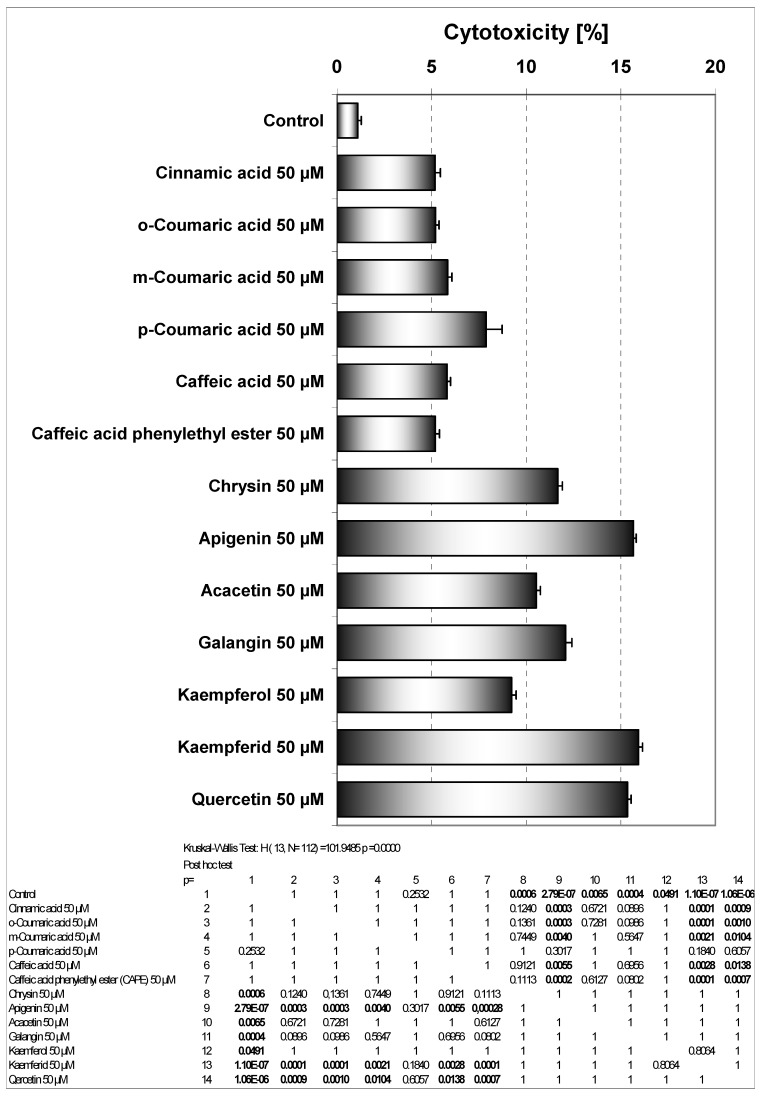
Cytotoxic activity of EEP phenolic components in HeLa cells. The cancer cells were incubated for 48 hours with the compounds at the concentrations of 50 μM. The percentage of death cells was measured by MTT cytotoxicity assay.

We investigated the cytotoxic effect of thirteen phenolic components of propolis: cinnamic acid, *o*-coumaric acid, *m*-coumaric acid, *p*-coumaric acid, caffeic acid, caffeic acid phenylethyl ester (CAPE), chrysin, apigenin, acacetin, galangin, kaempferol, kaempferid, quercetin on HeLa cancer cells. These compounds were detected and isolated after thin-layer chromatography (only qualitative analysis) from our EEP sample [13]. Cytotoxicity of phenolic acids and flavonoids at concentrations of 50 μM on HeLa cell line measured by the MTT assay are depicted in Figure 3. The strongest cytotoxic activity on HeLa cells was demonstrated by kaempferid (15.94±0.71%), apigenin (15.66±0.53%) and quercetin (15.36±0.66%). Among phenolic acids the greatest cytotoxic activity was associated with *para-* hydroxylation (*p*-coumaric acid). Apigenin and kaempferol exhibit similar B ring structures. The presence of a hydroxyl group in position 3 (kaempferol) decreased its cytotoxicity in comparison to apigenin. Methoxylation of the hydroxyl group in position 4’ in ring B, increased (kaempferid *versus* kaempferol) or decreased (acacetin *versus* apigenin) this activity. The presence of a hydroxyl group in position 3 decreased the cytotoxic effects only in case of a compound with q hydroxyl group in position 4’ (kaempferol *versus* apigenin). Activities of chrisin and galangin were similar. The higher activity of apigenin than kaempferol may be at least partially dependent on its ability to react with reactive oxygen species (ROS) that are important in induction of apoptosis. The hydroxyl group in position 3 of kaempferol is coupled with the carbonyl group in position 4 and the hydroxyl group in position 4’ and can determne its redox activity compared to apigenin where the hydroxyl group in position 4’ is coupled with a carbonyl group. Early investigation showed stimulation of luminol oxidation by hydrogen peroxide with horseradish peroxidase by apigenin and *p*-coumaric acid. The presence of a hydroxyl group in position 4’ (apigenin) stimulated the reaction, but a hydroxyl group in position 3 and a hydroxyl group in position 4’ (kaempferol) or two hydroxyl groups in the B phenyl ring and in position 3 (quercetin) inhibited this reaction. This reaction was inhibited by acacetin (-OCH_3_ in position 4’) and *o*- and *m*-coumaric acid [22]. The importance of hydroxyl groups in polyphenols has been underscored by other authors [23,24,25]. Also the cytotoxic effect of phenolic compounds may depend on lipophilicity that is very important for penetration into cells. The maximum solubility of apigenin in culture medium is smaller than that of kaempferol and quercetin [23]. On the other hand, lipids and proteins present in biological membranes facilitate the solubilization of polyphenols. Differences in cell membrane structures and metabolic activation of chemicals can also affect their activity. Similar cytotoxic activity of phenolic compounds has been reported by other authors [23,24,26].

**Figure 4:**
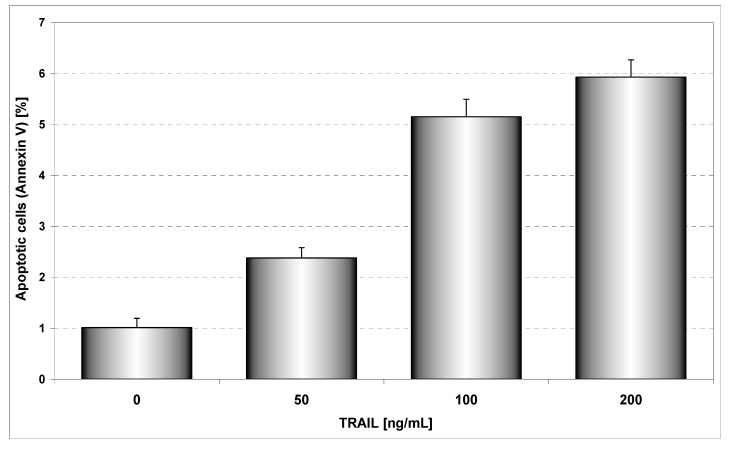
TRAIL induced apoptosis in HeLa cells. The cancer cells were incubated for 48 hours with TRAIL at the concentrations of 50 – 200 ng/mL. Detection of apoptotic cell death by annexin V-FITC staining using flow cytometry.

TRAIL induces programmed death in various cancer cells *in vitro* and *in vivo* [16]*.* However, some tumor cells are resistant to TRAIL-mediated cytotoxicity [17]. Figure 4 presents TRAIL induced apoptosis in HeLa cancer cells determined by annexin V staining followed by flow cytometry. The 48 hours’ exposure to TRAIL increased the percentage of apoptotic cells in a dose-dependent manner. TRAIL was less active against HeLa cells. For example, exposure to 100 ng/mL TRAIL induced apoptosis of 5.15±1.18% of cancer cells. We have confirmed that HeLa cells are resistant to TRAIL [27]. Previous reports have suggested that the sensitivity cancer cells to TRAIL-induced apoptosis can be correlated to the relative expression of death receptors TRAIL-R1 and TRAIL-R2 or intracellular levels of antiapoptotic protein (survivin, Bcl-xL, Bcl-2, inhibitor of apoptosis) [17].

TRAIL induced the cytotoxic effect in cancer cells on the apoptotic way. The necrotic cell death percentage of HeLa cells examined by Apoptest-FITC and lactate dehydrogenase assay was near 0%. The widespread occurrence of dysfunctions of the immune system require new approaches. Immunomodulation through natural or synthetic substances may be considered as alternative for the prevention and cure of infections or tumor disease [15]. Immunomodulatory effects of propolis and its phenolic components have been recorded. Macrophages, cytotoxic lymphocytes and NK cells play an important role in antitumor response. Treatment of mice with water extract of propolis modified tumoricidal activity of their immune cells [9,12,13,14,15]. 

**Figure 5:**
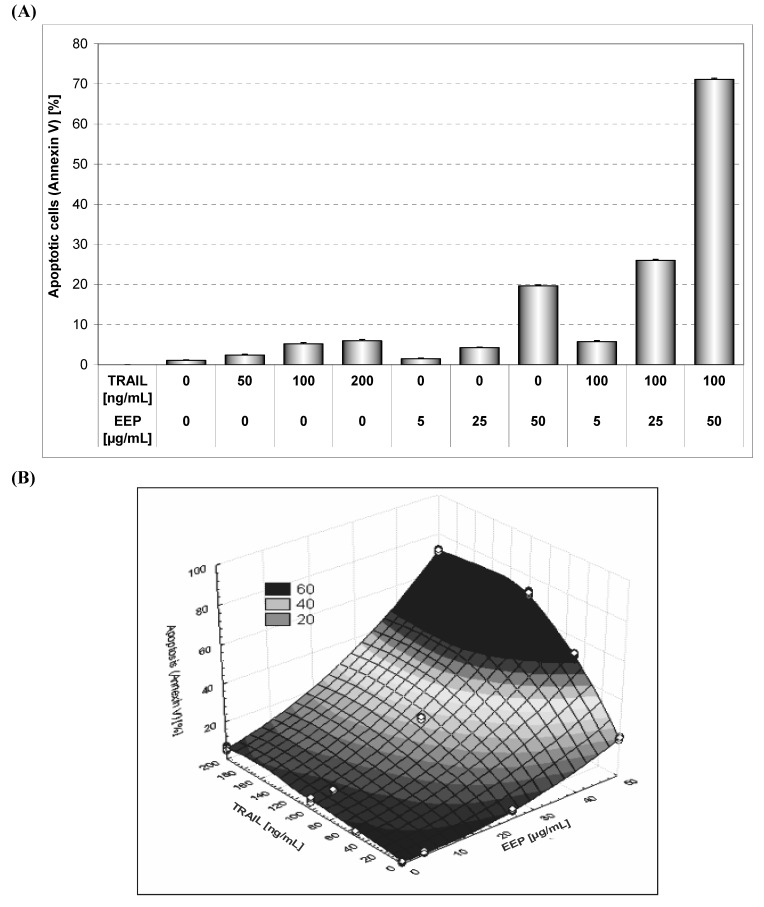
TRAIL induced apoptosis in combination with EEP in HeLa cells. Detection of apoptotic cell death by annexin V-FITC staining using flow cytometry. (A) The cancer cells were incubated for 48 hours with TRAIL at the concentration of 50 – 200 ng/mL and EEP at the concentration of 5 – 50 μg/mL and the cancer cells were incubated for 48 hours with TRAIL at the concentration of 100 ng/mL and EEP at the concentration of 5 – 50 μg/mL. (B) The same data was presented in 3-D figure.

Our study showed the impact of propolis and its phenolic compounds on the anticancer immune defense and the interaction between propolis or its bioactive constituents with TRAIL. As shown in Figure 2 and Figure 4, EEP or TRAIL alone induced little apoptotic effect on HeLa cells. We then tested EEP in combination with TRAIL on cancer cells. Surprisingly, we found that EEP strongly cooperated with TRAIL to induce apoptosis in HeLa cells. The percentage of the apoptotic cells after 48 hours’ exposure to 50 μg/mL EEP and 100 ng/mL TRAIL was elevated to 71.10 ± 1.16% (Figure 5). Propolis restored sensitivity of the tumor cells to TRAIL. For the first time our results demonstrated that EEP markedly augmented TRAIL mediated apoptosis in cancer cells. 

EEP enhanced the apoptosis-inducing potential of TRAIL and sensitized TRAIL-resistant HeLa cells. Further investigations will be required to explain the molecular mechanisms and cellular signaling pathways by which EEP sensitizes cancer cells to TRAIL induced death.

**Figure 6:**
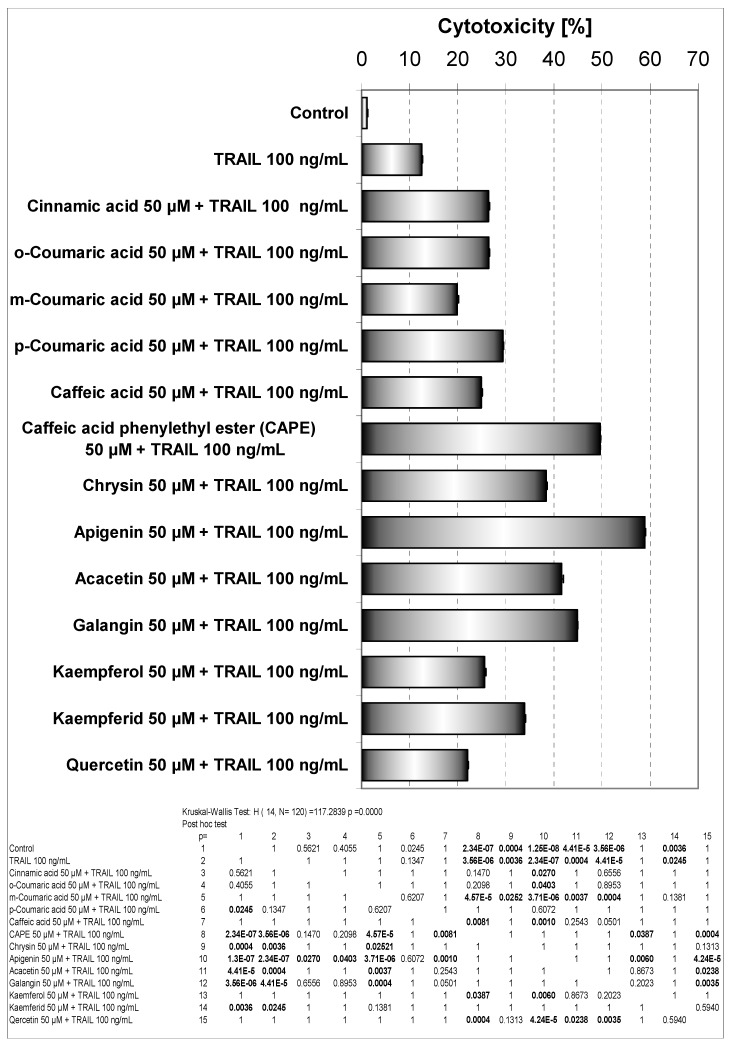
Cytotoxic activity of EEP phenolic components in combination with TRAIL in HeLa cells. The cancer cells were incubated for 48 hours with the compounds at the concentrations of 50 μM and TRAIL at the concentrations of 100 ng/mL. The percentage of cell death was measured by MTT cytotoxicity assay.

It has been suggested that a great deal of biological activities of propolis is mainly mediated by the presence of flavonoids and phenolic acids in it. These phenolic compounds can induce activities of the immune system and exert anitumor effects [9,11,13]. We investigated the cytotoxic effect of phenolic components of propolis in combination with TRAIL on HeLa cancer cells. All those compounds were detected in our sample of EEP [13]. Cytotoxic effect of phenolic acids or flavonoids combined with TRAIL in HeLa cell line measured by MTT assay is shown in Figure 6.

As shown in Figure 3, little cytotoxicity was observed by the phenolic components of propolis. Those phenolic compounds in combination with TRAIL increased the percentage of cell death compared to cytotoxicity of TRAIL alone. The phenolic acids and particularly flavonoids restored TRAIL sensitivity in TRAIL-resistant HeLa cells.

The therapeutic effects of propolis depend mainly on the presence of flavonoids. As reported, these polyphenols modulated the anticancer activity of the immune system [9,13,15]. In our study apigenin markedly augmented TRAIL mediated death of HeLa cells and exhibited the strongest cytotoxic effect in combination with TRAIL. The cotreatment of apigenin with TRAIL drastically caused an increase of cell death in Hela cells to 58.87±0.75%, compared to single agents. The activity of apigenin was associated with hydroxyl group in position 4’ and its ability to react with free radicals. Additionaly a hydroxyl group in position 3 (kaempferol) decreases cytotoxic effect. Increase of cytotoxicity in the presence of TRAIL and quercetin (two hydroxyl groups in ring B) was lower than in case of kaempferol.

A small number of similar studies with flavonoids in combination with TRAIL showed that apigenin, luteolin, quercetin and kaempferol synergistically induced apoptosis with TRAIL in human malignant tumor cells [20,27,28,29,30]. Horinaka *e*t *al.* reported that luteolin increased TRAIL-induced apoptosis in HeLa cells through upregulation of death receptor TRAIL-R2 (DR5) [27]. Horinaka *e*t *al*. in another investigation also showed enhancement of the apoptosis-inducing potential of TRAIL in DU145 prostate cancer cell line, Jurkat leukemic cell line and DLD1 colon cancer cell line. The combined use of apigenin and TRAIL caused Bcl-2-interacting domain cleavage, activation of caspases and increase expression of TRAIL-R2 [28]. Yoshida *et al.* determined that TRAIL-R2 upregulation by kaempferol augmented TRAIL action in colon cancer cells [29].

Kim *et al.* examined the molecular mechanisms by which quercetin augmented TRAIL-mediated apoptotic death in prostate cancer cells and confirmed the ability of quercetin to down-regulation of survivin expression. Among other inhibitors the authors added to cell as an inhibitor of extracellular – signal regulated protein kinase (ERK) PD98059, which is flavone derivative (2’-amino-3’-methoxy-flavone) and significantly maintained the intracellular level of survivin [30].

We also demonstrated for the first time that CAPE in combination with TRAIL enhanced the cytotoxic potential of the ligand (49.59±0.39% of cell death) and beside apigenin equally firmly sensitized TRAIL-resistant HeLa cells.

The previous study suggested that flavonoids increased expression TRAIL-R2. We hypothesize that propolis, as one of the richest sources of flavonoids such as apigenin, kaempferol or quercetin, could be a modulator of the expression of death receptors for TRAIL. Further investigation will be required to recognize and explain the molecular mechanisms by which EEP and its compounds act on cellular signaling pathways and sensitize cancer cells to TRAIL induced apoptosis.

The necrotic cell death percentage of HeLa cells incubated with EEP at the concentrations of 5 – 50 μg/mL or its phenolic components with or without TRAIL, examined by lactate dehydrogenase (LDH) leakage was near 0%.

**Figure 7:**
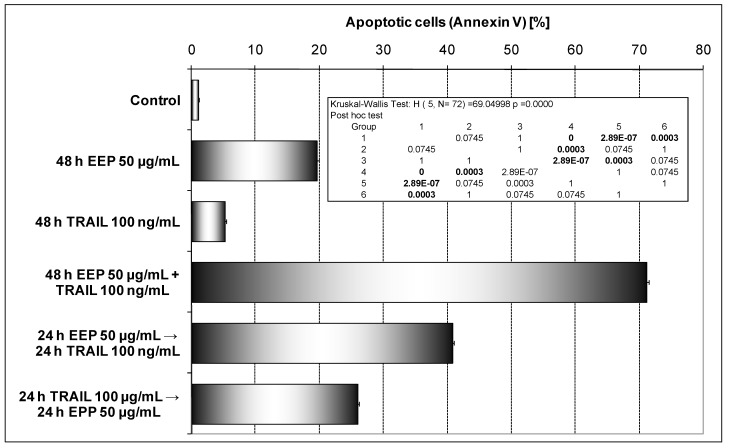
TRAIL induced apoptosis in combination with EEP, after and before exposure to EEP in HeLa cells. Detection of apoptotic cell death by annexin V-FITC staining using flow cytometry. HeLa cancer cells were: 1) treated with EEP in combination with TRAIL for 48 hours, 2) pretreated with EEP for 24 hours, followed by TRAIL for another 24 hours and 3) pretreated with TRAIL for 24 hours, followed by EEP for another 24 hours.

The sequence of drug administration is important to obtain maximum therapeutic benefits in a mixed therapy. We therefore examined weather cotreatment of HeLa cells with EEP and TRAIL induced greater apoptosis than the concurrent pretreatment with EEP followed by TRAIL and *vice versa* (Figure 7).

Interestingly, the cotreatment of HeLa cells with EEP in combination with TRAIL induced greater apoptosis than concurrent pretreatment or single agent alone. Reverse sequence of treatments: pretreatment EEP followed by TRAIL or pretreatment TRAIL followed by EEP has resulted in a significantly smaller apoptosis than in the cotreatment of EEP and TRAIL.

Among considered mechanisms of cell apoptosis in the presence of TRAIL and phenolic compounds or EEP are activation of caspase-8, -9, -3; MAPK (mainly ERK), *via* Bcl-2 family proteins, NF-κB and others [26,30].

Acacetin, a compound with lower activity than apigenin in our study, induced apoptosis by different routes e.g. activation of caspase cascades, ROS generation, mitochondria – mediated cell death signaling, the SAPK/JNK1/2 –c – Jun signaling pathway, p53, Fas/FasL apoptotic system and others in experiments on cancer cells performed by other authors [31,32,33,34,35].

We showed for the first time that EEP sensitizes cancer cells to TRAIL induced apoptosis. Our results indicated that EEP markedly augmented TRAIL mediated apoptosis in HeLa cells but further study will be required to examine the molecular mechanisms and cellular signaling pathways by which EEP and its compounds sensitize cancer cells to TRAIL induced apoptosis. The TRAIL-mediated cytotoxic and apoptotic pathways may be a target of the chemopreventive agents in human tumor cells and the overcome of TRAIL-resistance by propolis and its phenolic components may be one of the mechanisms responsible for their cancer preventive effects.

## 3. Conclusions

Dietary supplements, complementary or alternative to medication, become increasingly popular and it has been major interest in development of compounds of natural origin with chemopreventive properties. The emerging fields of cancer prevention by chemopreventive agents offer significant promise for reducing the incidence and mortality of cancer. Propolis is one of the richest sources of plant phenolics: flavonoids and phenolic acids, which are widely recognized as chemopreventive components. 

Our experiments showed that EEP sensitized TRAIL-resistant HeLa cells and augmented TRAIL induced apoptosis in cancer cells. The obtained results confirmed the significance of propolis in chemoprevention of malignant tumors. EEP as dietary supplement may be useful as a chemopreventive agent against cancer.

## 4. Experimental

### 4.1.Chemicals

#### 4.1.1 TRAIL

Recombinat human TRAIL was purchased from PeproTech Inc. (Rocky Hill, NJ, USA).

#### 4.1.2. EEP

Propolis was collected manually from the beehive of the Medical University of Silesia, and was kept desiccated pending its processing. It was extracted in 95% v/v ethyl alcohol, in a hermetically-closed glass vessel for 4 days at 37 °C, under occasional shaking. The ethanolic extract was then filtered through a Whatman filter paper No 4 and evaporated on a rotary evaporator, under reduced pressure at 60 °C. The same collection and extraction procedures were used throughout all our laboratory studies [13].

#### 4.1.3. Flavonoids and phenolic acids

Chrysin, apigenin, acacetin, galangin, kaempferol, kaempferid, quercetin, cinnanic acid, *o*-coumaric acid, *m*-coumaric acid, *p*-coumaric acid, caffeic acid, caffeic acid phenylethyl ester (CAPE) were purchased from Carl Roth GmbH (Karlsruhe, Germany) and Sigma Chemical Company (St. Louis, MO, USA). The reagents were dissolved in DMSO to a final concentration of 0.01% in media.

### 4.2. Cell culture

The experiments were performed on human cervical cancer HeLa cell line (DSMZ - Deutsche Sammlung von Mikroorganismen und Zellkulturen GmbH – German Collection of Microorganisms and Cell Cultures, Braunschweig, Germany). Cells were grown in monolayers cultures in RPMI 1640 medium (PAA – The cell culture company, Germany through Immuniq, Poland) containing 10% fetal bovine serum (FBS) (PAA – The cell culture company, Germany through Immuniq, Poland), 4 mML-glutamine (PAA – The cell culture company, Germany through Immuniq, Poland), 100 U/mL penicillin (Sigma Chemical Company (St. Louis, MO, USA), and 100 μg/mL streptomycin Sigma Chemical Company (St. Louis, MO, USA) and incubated at 37^o^C in atmosphere containing 5% CO_2_ [20].

### 4.3. Cytotoxicity assay

The cytotoxic effect of EEP and its phenolic compounds in combination with TRAIL on cancer cells was measured by the MTT (3-[4,5-dimethylthiazol-2-yl]-2,5-diphenyltetrazolium) assay as described [36]. HeLa cells were plated in a 96-well plate at a concentration of 2.5 x 10^5^/mL (5 x 10^4^/well) 24 h before the experiments. Various combinations of EEP, flavonoids and phenolic acids with or without TRAIL were added to the cells, and 48 h later the medium was removed and 20 μL MTT solutions (5 mg/mL) (Sigma Chemical Company, MO, USA) were added to each well for 4 h. The resulting crystals were dissolved in DMSO. Controls included native cells and medium alone. The spectrophotometric absorbance of each well was measured using a plate microreader with a test wavelength at 550 nm. The data on the cell proliferation and cytotoxic assays were obtained from three separate experiments and for each test quadruplicate wells were used. The percent of cytotoxicity was calculated by the formula, percent cytotoxicity (cell death) = (1-[absorbance of experimental wells/absorbance of control wells]) x 100.

### 4.4. LDH (lactate dehydrogenase) release assay

LDH is a stable cytosolic enzyme that is released upon membrane damage. LDH activity was measured using a commercial cytotoxicity assay kit (Roche Diagnostics GmbH, Mannheim, Germany), in which released LDH in culture supernatants is measured with a coupled enzymatic assay, resulting in conversion of a tetrazolium salt into red formazan product. The cells were treated with various concentrations of EEP, its phenolic compounds alone and in combination with TRAIL for indicated period of time. The sample solution (supernatant) was removed and LDH released from cells was measured in culture medium. The maximal release was obtained after treating control cells with 1% Triton^®^ X-100 (Sigma Chemical Company, St. Louis, MO) for 10 minutes at room temperature [37]. The necrotic percentage was expressed using the formula, (sample value/maximal release) x 100%. All experiments were done in triplicate.

### 4.5. Determination of apoptotic cell death by annexin V-FITC staining

Apoptosis was measured using flow cytometry to quantify the levels of decentable phosphatidylserine (PS) on the outer membrane of apoptotic cells. Externalized PS on the outer surface of the cytoplasmic membrane becomes labeled by fluorescein-labeled annexin V, which has a high affinity for PS-containing phospholipid bilayers [38]. HeLa cells (2.5 x 10^5^/mL) were incubated for 48 h with EEP and/or TRAIL and then washed twice with PBS and resuspended in 1 mL of binding buffer. Five hundred microliters of cell suspension was then incubated with 5 μL of annexin V-FITC and 10 μL of propidium iodide (PI) for 10 min at room temperature in the dark. The annexin V assay was performed using the Apoptotest-FITC Kit (Dako, Glostrup, Denmark). The population of annexin V-positive cells was evaluated by flow cytometry (BD FACScan, BD Bioscences, San Jose, CA, USA). The Apoptest-FITC was performed in triplicate.

### 4.6. Statistical analysis

The results are expressed as means ±S.D. obtained from three separate experiments. The experimental means are compared to the means of untreated cells harvested parallel and the data is polled for replicate experiments. Statistical significance was evaluated using one- and multiple-way ANOVA Kruskal-Wallis test followed by the Levene test. *P*-values less than 0.05 were considered significant.
